# Neonatal diabetes in Wolcott–Rallison syndrome: a case report

**DOI:** 10.1186/1687-9856-2013-S1-P4

**Published:** 2013-10-03

**Authors:** Can Thi Bich Ngoc, Vu Chi Dung, Sarah Flanagan, Sian Ellard

**Affiliations:** 1 National Hospital of Pediatrics, Hanoi, Vietnam; 2 Royal Denvon and Exter NHS Healthcare Trust, Exeter, UK

## 

Wolcott-Rallison syndrome (WRS) is a rare autosomal recessive disorder characterized by the association of permanent neonatal or early-infancy insulin-dependent diabetes, multiple epiphyseal dysplasia and growth retardation, and other variable multisystem clinical manifestations. In the present study, we analyzed the EIF2AK3 gene in a 64 day-old-girl WRS patient and his parents to study the clinical features, the mechanism for genetic onset of WRS and provide credible genetic counseling for prenatal diagnosis in his family. Based on analysis of a 64 day-old-girl’s clinical symptoms associated with biochemical examination, the diagnosis of WRS was therefore made. Genomic DNAs were extracted from peripheral blood leukocytes from the patient and her parents with their informed consent for genetic studies. The coding and flanking intronic regions of the EIFAK3 gene was analysed by sequencing. In a result, the patient had gestation age of 41 weeks, birth weight of 3200 g, and onset of the disease at 64 days of age. She was admisted with the features of convulsion, anemia, jaundice, diabetic ketoacidosis with pH of 7.27, HCO_3_^-^ of 17.8 mmol/l, BE of -8 mmol/l, blood glucose 42.46 mmol/l, HbA1C 6.5 %, total bilirubin 59.2µmol/l, direct bilirubin 29.7 µmol/l, AST 3741.2 U/l, ALT 1927 U/l. PCR of CMV, EBV, HAV were negative. Abdominal ultrasound did not find any sign of cholestasis. Sequencing analysis of patient’s EIF2AK3 gene has identified a homozygous missense mutation, p.R632W. The parents are carriers of heterozygous EIF2AK3 missense mutation, p.R632W. In conclusion, combining mutation screening of EIF2AK3 gene with clinical manifestations and effective examination may provide a reliable diagnostic method for patients.

